# Enhancing cognition before clinical symptoms of dementia

**DOI:** 10.3389/fnsys.2014.00240

**Published:** 2014-12-16

**Authors:** Rafael Franco

**Affiliations:** ^1^Department of Biochemistry and Molecular Biology, Faculty of Biology, University of BarcelonaBarcelona, Spain; ^2^CIBERNED: Centro de Investigación en Red sobre Enfermedades Neurodegenerativas, Instituto de Investigación Carlos IIIMadrid, Spain

**Keywords:** Alzheimer's, ascorbic acid, dietary supplement, epigenetic, histone deacetylase, clinical trial, phosphodiesterase inhibitor, tadalafil

As the title of the special issue indicates, controversy surrounds augmentation of brain cognition in humans. Lacking efficacious drugs for Alzheimer's disease (AD) and with many AD patients recruited for clinical trials that unfortunately do not provide the expected results, one wonders whether to test cognition enhancement strategies in individuals without symptoms of cognition decline. This opinion article presents the view that safe drugs and or dietary supplements should be tested worldwide in aged individuals under the control of a non-for-profit organization.

Unfortunately, the effort to translate the results in rodents into patients with dementia, mainly of the Alzheimer's type has not provided the expected results. The reasons for the loss-in-translation are varied (see Franco and Cedazo-Minguez, 2014). Moreover, failures on achieving efficacious anti-AD medications and the high cost of performing clinical trials make pharmaceutical companies to abandon the dementia field (see http://www.abc.net.au/pm/content/2012/s3611062.htm). Clinical trials face the difficulty of patient recruitment and the need—due to ethical reasons- to maintain the already approved anti-AD medication. It is difficult to attain the primary outcomes in AD patients under a multi-drug treatment regime. An alternative approach consists of testing cognition enhancement in individuals not taking anti-AD medication, even in those without any clinical symptom of dementia.

A controversy concerning supplements of vitamin D in individuals with little or no clinically-relevant symptoms attracted enough interest to allocate one discussion session in the 15th European Congress of Endocrinology held in Copenhagen in 2013. A similar controversy on testosterone supplementation exists among endocrinologists and nutritionists. Solid reasons emerge for and against the convenience of those supplementations; yet these compounds are easily available. On analyzing the benefit-risk balance, the main concern is the side effects that may appear after chronic treatment with vitamin D or testosterone. A similar concern arises on thinking about the possibility to prescribe cognition enhancers under a chronically regime. Research in animal models clearly indicates that cognition enhancement is possible. Should drugs with cognition-enhancing potential in mice models of dementia be tested in healthy humans? I consider, for instance, that safe drugs deserve a chance to be tested in aged non-demented humans.

Relevant for the present discussion is that drugs may be prescribed to individuals without any clinical symptom. Statins, which are inhibitors of 3-hydroxy-3-methylglutaryl coenzyme A reductase, are instrumental for the prevention of cardiovascular dysfunction in hypercholesterolemic patients. Statins are taken in familial hypercholesterolemia at relatively high doses, chronically and from very early in life. Statins are safe as deduced from the records of millions of patients taken the medication since 1985, when the first statin was available for human use. Statin development was on the verge to be abandoned due to potential side effects of blood lipid-lowering drugs. An important pharmaceutical company took the decision of discontinuing statin development. Relevant here is that the “*FDA*—food and drug administration—*became actively involved in maintaining interest in the development of the statins*.” Also relevant is that “*there was no proof at that time—*early eighties*—that drugs or diet used to lower cholesterol would be the clinical equivalent of patients with spontaneously occurring low cholesterol*,” meaning that statins were being developed without the certainty that lowering cholesterol by statins could be efficacious in combating atherosclerosis. The full drug development story is available at http://www.fda.gov/AboutFDA/WhatWeDo/History/ProductRegulation/SelectionsFromFDLIUpdateSeriesonFDAHistory/ucm082054.htm. In summary, safe and efficacious statins are prescribed even in the absence of clinical symptoms.

Interestingly, some compounds approved for non-CNS indications have shown cognition enhancing properties in animal models of AD. As scientist in a laboratory on translational AD research I had experience on two drugs with good safety records: 4-phenylbutyrate (PBA) and tadalafil. The first is used in children with thalassemia, sickle-cell disease or congenital defects in enzymes of the urea cycle (Dover et al., 1994; Collins et al., 1995; Maestri et al., 1996). The second is one of the phosphodiesterase V inhibitors prescribed in erectile dysfunction (Boolell et al., 1996a,b) and pulmonary hypertension (Prasad et al., 2000; Weimann et al., 2000; Ghofrani et al., 2004; Kukreja et al., 2004; Affuso et al., 2006). Two are the mechanisms underlying the cognition-enhancing effects of PBA in mouse models of AD (reviewed in Cuadrado-Tejedor et al., 2011a, 2013). On the one hand, PBA is a chemical chaperone that may help in preventing the formation of protein aggregates. On the other hand, PBA is a histone deacetylase inhibitor that enhances the transcription of genes involved in memory processes. Inhibition of phosphodiesterase V is effective in AD models by increasing the concentration of cGMP that in turn enhances the neural mechanisms of defense against tau hyperphosphorylation and Aß aggregation (Rutten et al., 2007; Puzzo et al., 2009; Cuadrado-Tejedor et al., 2011b, 2013; Zhang et al., 2013; García-Barroso et al., 2013, 2014). Importantly, whereas acute treatment did not affect cognitive performance in healthy individuals (Reneerkens et al., 2013), a double-blind placebo-controlled study in patients with erectile dysfunction showed cognitive enhancement of a chronically administered phosphodiesterase inhibitor (Shim et al., 2014). Even in a chronic regime phosphodiesterase V inhibitors are safe (Montorsi et al., 2004; Giuliano et al., 2010; www.fda.gov/drugs/drugsafety/ucm390876.htm). To bridge the gap of successful “anti-AD” therapies in mice that do not reach humans (see Franco and Cedazo-Minguez, 2014), safe drugs could, in my opinion, be the first to test.

I consider that a non-for-profit organization such as the US FDA may take the lead to select 3–4 safe drugs, a couple of dietary supplements and a placebo, and design a longitudinal study that should start in late middle-aged healthy individuals with a lifelong follow-up. Such a study will take long to give results, but they would be quite robust and of better quality as time passes. In summary my opinion is that cognitive enhancers in the form of safe drugs and safe dietary supplements (vitamin C for instance^1^) should be tested already in individuals without clinical symptoms of dementia. Less informative but easier to implement would be to follow the age-dependent cognitive decay in patients taking already chronic medication for non-CNS indications that has shown (in mice) cognitive enhancing potential. Such studies would benefit of the most recent criteria of dementia/AD diagnosis, of novel behavioral tests and of novel biomarkers of preclinical phases of AD (Jack et al., 2011; Sperling et al., 2011). Likely, the results would indicate the potential of the assayed drugs for improving cognition and/or delaying onset of dementia, and the convenience or, otherwise, the inconvenience of starting medication in apparently healthy individuals. Even individuals not entering into dementia might benefit from reducing the cognitive decline due to aging.

## Conflict of interest statement

The author is co-inventor in the patent application: TADALAFIL FOR THE TREATMENT OF DEMENTIA. PCT/EP2012/061234; Date: 06/13/2012. WIPO Patent Application WO/2012/171974 Kind Code: A1. The patent is abandoned as it did not enter into National phases.

## Footnotes

^1^A recent report shows positive results of vitamin C in both wild type and the APP/PSEN1 animal model of AD (Kennard and Harrison, 2014).
